# Study on the Application of Indocyanine Green Lymphography in the Management of Microcystic Lymphatic Malformation: A Randomized Clinical Trial

**DOI:** 10.3390/children13050584

**Published:** 2026-04-23

**Authors:** Tao Han, Yuan Wang, Jiageng Xiong, Jie Cui, Weimin Shen

**Affiliations:** Department of Burns and Plastic Surgery, Children’s Hospital of Nanjing Medical University, Nanjing 210008, China

**Keywords:** microcystic lymphatic malformation, indocyanine green, lymphography, treatment, children

## Abstract

**Objective:** To explore the application value of indocyanine green lymphography (ICGL) in the treatment of microcystic lymphatic malformation (mLM). **Methods:** This study was a prospective, randomized controlled clinical trial. Children with mLM who presented between November 2023 and November 2025 were recruited as subjects. They were randomly assigned in a 1:1 ratio to two groups: experimental group (ICGL-guided partial resection of mLM + penetration sclerotherapy), or control group (conventional partial resection of mLM + penetration sclerotherapy). Clinical baseline data were recorded, including gender, age, lesion location, and maximum lesion diameter. The primary outcomes were efficacy-related parameters (cure rate, effective rate, and number of subsequent treatments). Secondary outcome measures included intraoperative and postoperative parameters (operative time, postoperative drainage, follow-up duration) and related complications. **Results:** A total of 39 children completed the treatment and follow-up and were included in the final analysis (20 in experimental group and 19 in control group). The baseline characteristics were balanced and comparable between the two groups (*p* > 0.05). Regarding the primary outcomes, efficacy analysis revealed that the cure rate was significantly higher in the experimental group than in the control group (65.0% vs. 26.3%, *p* < 0.05). However, there were no significant differences between the groups in effective rate (experimental group: 95.0% vs. control group: 89.5%) or the number of cases requiring subsequent treatment (experimental group: 2 cases vs. control group: 3 cases) (*p* > 0.05). Furthermore, no statistical difference was observed between the two groups in secondary outcomes, including operative time, postoperative drainage, follow-up duration, or the incidence of postoperative complications (*p* > 0.05). **Conclusions:** ICGL-guided partial resection of mLM + penetration sclerotherapy improves postoperative outcomes in mLM. By enabling real-time intraoperative assessment of mLM lesion extent, this strategy facilitates resection of the main lesion and precise sclerotherapy of residual microcysts. These findings validate the significant application value of the ICGL in personalized treatment for mLM.

## 1. Introduction

Cystic lymphatic malformation (cLM) is a common congenital, benign vascular anomaly in children, characterized by malformed and aberrantly dilated lymphatic vessels [1]. The conventional therapeutic strategy for cLM is primarily determined based on factors such as lesion location, size, and histological subtype [2,3]. Among these, microcystic lymphatic malformation (mLM), characterized by tiny cystic cavities with a volume of <2 cm^2^, presents a persistent challenge in current management due to its infiltrative growth pattern, ill-defined borders, and relatively solid consistency [4,5]. In contrast to the macrocystic and mixed-cystic types, which demonstrate well-established therapeutic efficacy, mLM generally responds poorly to conventional sclerotherapy. This often manifests as repeated treatments, higher recurrence rate, and greater tendency to induce excessive proliferation of surrounding tissues, consequently impacting both esthetics and local function [6,7]. Surgical resection, as one of the treatment modalities for mLM, is encumbered by a series of inherent and difficult-to-overcome limitations. Firstly, due to the diffuse and infiltrative growth of microcystic lesions, a “radical resection” is unattainable [8,9]. Secondly, even when a “debulking surgery” is performed, residual microcysts within the peripheral tissues cannot be precisely identified, potentially leading to postoperative recurrence [3,10]. Consequently, combined treatments integrating surgery, sclerotherapy, and oral sirolimus are gradually being employed to improve postoperative outcomes for mLM.

Currently, preoperative imaging modalities (ultrasonography [US], computed tomography [CT], or magnetic resonance imaging [MRI]) primarily provide static morphological information, but fail to reveal real-time distribution range of microcystic lesions and their connection with lymphatic draining system [11]. This limitation often renders the intraoperative determination of sclerotherapy and resection margins [12]. Ros et al. described a lymphographic-like technique involving the slow infusion (flow rate of 0.7 mL/h) of a bleomycin-contrast agent mixture into microcystic cavities, utilizing intraoperative low-dose CT scan to check diffusion of the mixture in mLM [13]. Alternatively, Ding et al. performed DSA lymphangiography via inguinal lymph node puncture to delineate the extent of microcystic lesions within the vagina and pelvis [14]. However, the associated risk of radiation exposure limits the wide application of such lymphography in the pediatric mLM patients.

Indocyanine green (ICG) is a highly water-soluble, iodinated tricarbocyanine dye presenting as a dark green, loose, solid powder. As it is readily degraded by visible light and heat, it must be stored at low temperature (−20 °C) protected from light. Prior to use, ICG is dissolved in sterile water for injection (concentration: 2.5 mg/mL). After injection into the body, ICG rapidly binds non-specifically to proteins in the blood or lymph fluid, enabling its use for imaging of blood vessels and lymphatic vessels. Upon excitation with near-infrared laser irradiation, the fluorescent signal can penetrate tissue up to approximately 2 cm in thickness and is captured by an infrared camera to generate dynamic images. ICG fluorescence imaging is widely used for monitoring flap blood supply, delineating tumor boundaries, assessing liver function, and localizing sentinel lymph nodes, owing to its favorable tissue penetration, rapid metabolism, low toxicity, and non-radioactive nature [15,16]. Notably, indocyanine green lymphography (ICGL) has been extensively applied in the diagnosis and treatment of primary or secondary lymphedema. This imaging technique provides a scientific basis for assessing lymphatic disorders, evaluating structural and functional alterations in lymphatic vessels, and guiding the reconstruction of lymphatic drainage [17]. Our previous research had revealed that in stage I/IIA mLM of the tongue, the characteristics observed under ICGL was the presence of numerous tortuous and interwoven lymphatic inflows within the lesion, forming a diffuse fluorescence pattern. Consequently, this unique imaging manifestation allowed a precise definition of lesion margins and enhanced intraoperative decision-making [18].

To date, most existing evidence is limited to case series or retrospective studies, lacking prospective comparative data. Therefore, the optimal surgical strategy for mLM remains controversial, and the additive value of ICG navigation over conventional treatment has not been rigorously tested. In the present study, we designed a prospective, randomized controlled trial aimed at evaluating safety, feasibility, and curative effects of the strategy “ICGL-guided partial resection + penetration sclerotherapy” in the management of mLM. This research will not only explore a novel effective strategy for mLM but also further validate the clinical value of precise intraoperative intervention guided by ICGL.

## 2. Patients and Methods

### 2.1. Study Patients

This prospective, randomized controlled clinical study was conducted at our center from November 2023 to November 2025, enrolling pediatric cases with mLM. Our study was approved by the Ethics Committee of Children’s Hospital of Nanjing Medical University (202311019-2) and was registered at https://clinicaltrials.gov/ (NCT06275022). This research was conducted and reported in accordance with the CONSORT guidelines for randomized trials. The guardians of eligible children meeting the inclusion criteria received comprehensive information and provided written informed consent prior to enrollment. The clinical data collected in this study encompassed baseline information (gender, age, histological subtype, location, maximum lesion diameter), perioperative parameters, clinical outcomes, and postoperative complications. All cases were diagnosed as mLM based on preoperative physical examination, imaging assessment (including US and MRI), and postoperative pathology.

The inclusion criteria included the following: (1) no previous intervention; (2) diagnosis of mLM; (3) lesion confined to one single body region (cervicofacial region, extremities, or trunk); (4) patients with an ability to participate in or follow-up during the study. The exclusion criteria included the following: (1) deep lesions (muscle involvement); (2) coexistence of other types of vascular malformations; (3) history of iodine allergy; (4) severe liver or kidney dysfunction. Withdrawal and termination criteria: (1) voluntary withdrawal of the participant during the study; (2) postoperative pathological findings indicating other types of vascular malformations; (3) loss to follow-up postoperatively.

### 2.2. Randomization

The participants in this study were divided into two groups: the experimental group (ICGL-guided partial resection of mLM + penetration sclerotherapy) and the control group (conventional partial resection of mLM + penetration sclerotherapy). A complete randomization method was employed for group allocation, with the following steps: Firstly, patients were consecutively numbered according to their visit order. Subsequently, a random number sequence was generated using SPSS Statistics 22.0 software (IBM Corp., Armonk, NY, USA), and eligible patients with the sequence were randomly assigned at a 1:1 ratio to the experimental group or control group. Group assignments were placed in sequentially numbered, opaque, sealed envelopes. After a patient consented and was deemed eligible, the attending surgeon opened the next envelope to reveal the allocation. Surgeons and patients were not blinded due to the use of ICG fluorescence, but outcome assessors were blinded to group assignment.

### 2.3. Study Intervention

**Figure 1:**
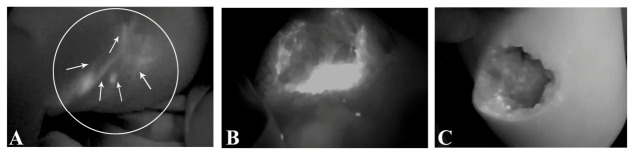
Illustration of ICGL-guided partial resection of mLM. (**A**) ICGL revealed tortuous and slender lymph vessels within the lesion, exhibiting a diffuse distribution. (**B**) Delineation of the main lesional extent through the incision. (**C**) Identification of residual microlesions at the periphery based on the fluorescent signal following partial resection of the lesion. ICGL, indocyanine green lymphography; mLM, microcystic lymphatic malformation. *Arrow head*, lymph vessel; *circle*, region of the lesion.

In the experimental group (ICGL-guided partial resection of mLM + penetration sclerotherapy), under general anesthesia, a total of 0.1 mL of ICG solution (2.5 mg/mL; Dandong Yichuang Co, Ltd., Dandong, China) was administered via multi-point intradermal injections distal to mLM lesion. The maximum dosage per session was limited to 0.5 mg/kg. After 10 min localized massage, ICGL was conducted using a near-infrared (NIR) fluorescence imaging system (Mingde Medical Diagnosis Inc., Langfang, China). This imaging modality was employed to evaluate lymphatic drainage patterns and mLM lesion borders in real time. The ICG injection points for mLM at specific anatomical regions were summarized in our previous study [19]. The extent of mLM lesion was assessed based on the diffusely distributed ICG fluorescent signal. Subsequently, a skin incision was designed on the lesion surface or at a lower-positioned concealed site. Following incision, the subcutaneous extent of the lesion was confirmed under ICGL guidance. While preserving the surrounding vessels and nerves, the majority of mLM tissue was resected as extensively as possible. Following resection, residual ICG-positive microlesions were thoroughly penetrated (Figure 1). After aspirating the lymphatic fluid from the lesion, the cyst cavity was cauterized twice with 10 to 20 mL 2% iodine tincture (20 mL; Guangdong South China Pharmacy Co, Ltd., Zhanjiang, China). Following complete hemostasis, an intracystic negative-pressure drainage tube was placed, and the incision was closed.

In the control group (conventional partial resection of mLM + penetration sclerotherapy), a skin incision was made on the lesion surface or at a lower-positioned concealed site. Intraoperative gross assessment and subjective experience determined the extent of surgical resection, and the peripheral microlesions were penetrated to the extent determined by preoperative MRI. After thoroughly aspirating the lymphatic fluid, the cystic cavity was cauterized twice with 10 to 20 mL 2% iodine tincture. The incision was then closed after placement of intracystic negative-pressure drainage tube.

For both the experimental group and control group, all cases underwent sequential sclerotherapy postoperatively: 4–6 mL of polidocanol solution (3%; Hameln Pharmaceutical Gmbh Co., Ltd., Wiesbaden, Germany) was injected into the cystic cavity through the drainage tube, followed by gentle massage of the lesion to ensure even distribution of sclerosing agent. The polidocanol was retained for one hour and subsequently aspirated by reconnecting the drainage tube to negative pressure. This lavage procedure could be repeated every other day until the drainage volume had essentially ceased. Thereafter, an injection of pingyangmycin (8 mg; Laiboten Pharmaceutical Co, Ltd., Harbin, China) was administered before the removal of the drainage tube. The amount of bleomycin injection was 0.5 mg/kg, and the maximum dose was limited to 8 mg per session.

### 2.4. Evaluation of Curative Effects [20]

Postoperative assessment of curative effects was conducted through physical examination and imaging findings (US or MRI) in the modified intention-to-treat population of cases who underwent randomization. For imaging assessment, the size of each mLM lesion was measured independently by two radiologists who were blinded to group allocation. On either US or MRI, the longest diameter and its longest perpendicular diameter were measured, and the product of these two diameters (length × width) was used as the size index. The percentage reduction in size was calculated as: (preoperative size index—postoperative size index)/preoperative size index × 100%. The final percentage reduction used for curative effect evaluation was the average of the two radiologists’ measurements. Treatment response was evaluated as follows: (1) excellent, defined as >90% reduction in size; (2) good, 75–90% reduction; (3) fair, 50–75% reduction; and (4) poor, <50% reduction. The cure rate was defined as the percentage of patients who achieved excellent curative effect relative to the total number of cases; the effective rate was defined as the percentage of patients who achieved excellent, good, or fair curative effect relative to the total number of cases.

### 2.5. Statistical Analysis

This study employed a superiority design aiming at comparing the curative effect between two groups: the experimental group (ICGL-guided partial resection of mLM + penetration sclerotherapy) and the control group (conventional partial resection of mLM + penetration sclerotherapy), with the cure rate as the primary outcome measure. Based on findings from a previous study [21], where the cure rate following conventional sclerotherapy was approximately 35.1%, it was hypothesized that the experimental group would be superior to the control group. The sample size was calculated using a one-sided α level of 0.05 and approximately 80% power, assuming an expected difference in cure rates between the two groups. The sample size was calculated with a 1:1 allocation ratio using PASS 15.0 software (Stata Corp., LP, TX, USA), which determined a minimum requirement of 20 cases per group. Accounting for a dropout rate of approximately 15%, a sample size of 24 patients was required for each group in this study.

Data were expressed as numbers (percentages), means (SDs), and medians (interquartile ranges) as appropriate. Analyses of treatment effects were performed according to the modified intention-to-treat principle. Categorical data were assessed with Fisher’s exact test. The normality of distribution was assessed using the Shapiro–Wilk test for all the variables in this study, and continuous variables were compared by using independent two-sample *t*-tests or Mann–Whitney *U* tests, as appropriate. There were no missing data for both primary outcome and secondary outcome in the modified intention-to-treat population. The study was conducted in accordance with the CONSORT 2010 statement. Statistical analyses were conducted using SPSS Statistics 22.0 software. *p* value < 0.05 were considered statistically significant.

## 3. Results

### 3.1. Participants

**Figure 2:**
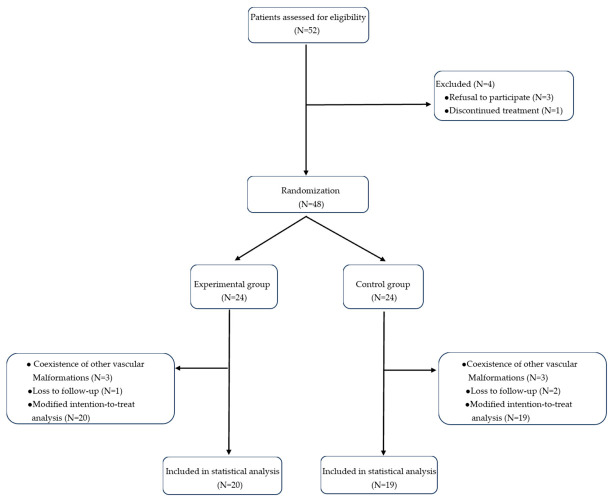
Flowchart of patient screening.

A total of 52 cases were assessed for eligibility in this prospective study, of whom 4 were excluded due to refusal to participate or discontinued treatment, resulting in 48 patients being randomized (Figure 2). Participants were allocated randomly in a 1:1 ratio to either the experimental group (N = 24) or control group (N = 24). During the study period, 4 cases in the experimental groups and 5 cases in the control group were withdrawn due to coexistence of other vascular malformations or loss to follow-up. Consequently, the included cases were randomized to the experimental group (N = 20) and control group (N = 19).

### 3.2. Baseline Information

The overall cohort exhibited a near-equal sex distribution (20 males, 51.3%; 19 females, 48.7%), with ages ranging from 1 month to 15 years. No statistical differences were observed between the two groups in terms of gender (*p* = 0.752), age (*p* = 0.748), lesion location (*p* = 0.667), or maximum lesion diameter (*p* = 0.320), confirming balanced baseline data and comparability (Table 1).

### 3.3. Primary Outcome

In relation to the primary outcome, the cure rate in the experimental group (65.0%, 13/20) was significantly higher than that in the control group (26.3%, 5/19). The effective rates were comparable between the two groups (95.0% in the experimental group vs. 89.5% in the control group), showing no statistically significant difference (*p* > 0.05). Subsequent sclerotherapy was administered to 2 patients in the experimental group and 3 patients in the control group, with no significant difference observed between the groups (*p* > 0.05). It is noteworthy that in one case with poor curative effect from the experimental group, subsequent treatment with both oral sirolimus and compression therapy was required to manage mLM progression (Table 2).

### 3.4. Secondary Outcome

**Figure 3:**
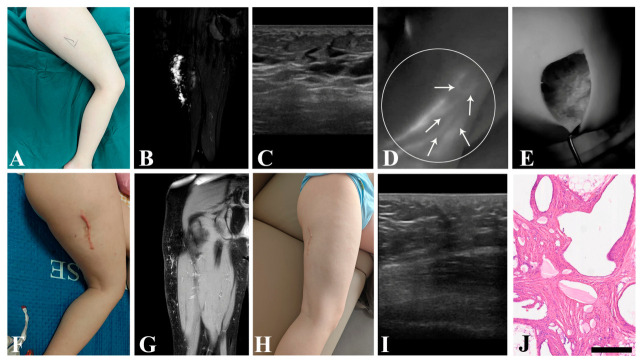
A 7-year-old male patient suffered from mLM of right thigh. (**A**) Preoperative appearance. (**B**) Preoperative MRI revealed microcystic lesions on the lateral aspect of the right thigh. (**C**) Preoperative US showed scattered small cystic cavities. (**D**) ICGL demonstrated multiple slender lymph inflows within the lesion, exhibiting a diffuse reticular distribution. (**E**) After resection of mLM lesion, the surgical field was re-examined under ICGL to identify residual microcysts. (**F**) Appearance at 6 months postoperatively. (**G**) MRI at 6 months postoperatively. (**H**) Appearance at 14 months postoperatively. (**I**) US at 14 months postoperatively. (**J**) Postoperative pathology. mLM, microcystic lymphatic malformation; MRI, magnetic resonance imaging; US, ultrasound; ICGL, indocyanine green lymphography. *Arrow head*, lymph vessel; *circle*, region of the lesion; scale bar = 500 μm.

**Figure 4:**
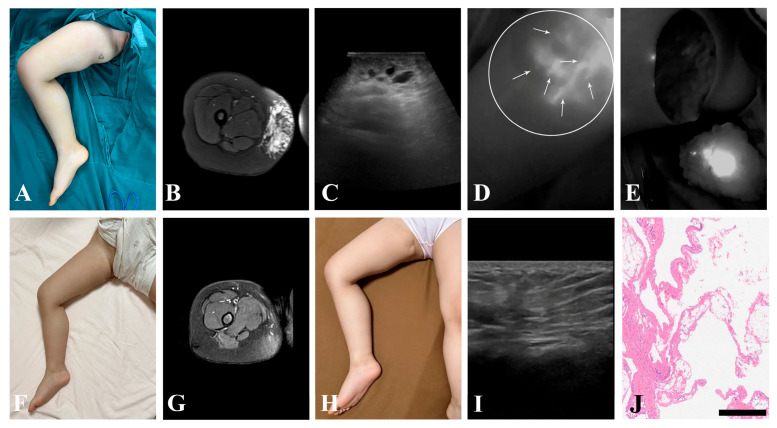
A 3-year-old female patient suffered from mLM of right thigh. (**A**) Preoperative appearance. (**B**) Preoperative MRI revealed microcystic lesions on the medial aspect of the right thigh. (**C**) Preoperative US showed scattered microcysts. (**D**) ICGL demonstrated multiple diffuse lymph inflows within the lesion. (**E**) After resection, the surgical field was re-examined under ICGL to identify residual microcysts. (**F**) Appearance at 4 months postoperatively. (**G**) MRI at 4 months postoperatively. (**H**) Appearance at 8 months postoperatively. (**I**) US at 8 months postoperatively. (**J**) Postoperative pathology. mLM, microcystic lymphatic malformation; MRI, magnetic resonance imaging; US, ultrasound; ICGL, indocyanine green lymphography. *Arrow head*, lymph vessel; *circle*, region of the lesion; Scale bar = 500 μm.

Comparisons of peri- and postoperative parameters are presented in Table 3. Although the use of intraoperative ICGL prolonged the operative duration in the experimental group (50.00 ± 15.05 min vs. 41.53 ± 14.60 min), this difference did not reach statistical significance (*p* = 0.091). Due to the inability to achieve complete blockage of all lymph inflow in mLM lesion, no significant difference was observed in postoperative drainage volume between two groups (*p* = 0.383). Furthermore, there was no significant difference in follow-up period comparison (*p* = 0.681). Typical cases are illustrated in Figure 3 and Figure 4.

Postoperative complications observed in this study included fever, pigment, abnormal hair growth, local infection, subcutaneous induration, and scar hyperplasia (Table 4). Fever occurred in 3 patients in the experimental group and 1 patient in the control group. One case of local infection was documented in each group. Pigmentation and subcutaneous induration showed gradual resolution during the 3 to 6 months postoperatively. Abnormal hair growth at the surgical site was observed in 5 patients (3 in experimental group and 2 in control group). All cases of scar hyperplasia demonstrated improvement after anti-scarring therapy. Overall, no statistical differences were found between the two groups regarding the incidence of various postoperative complications (*p* > 0.05).

## 4. Discussion

mLM is composed of numerous tiny cysts, characterized by diffuse and infiltrative growth pattern with ill-defined boundaries [22,23]. As the first-line therapeutic option, the efficacy of sclerotherapy relies on the homogeneous distribution and adequate retention of the sclerosant agent within the lesion [24,25]. However, within the sponge-like structure of mLM, the injected sclerosant fails to diffuse extensively, thereby hindering the achievement of satisfactory outcomes [26]. A study by Cha et al. demonstrated that multi-point, slow infusion of bleomycin using an syringe pump was a feasible approach for treating mLM, achieving a cure rate of up to 39% [27]. Overall, sclerotherapy alone for mLM is associated with a high recurrence rate, often necessitating repeated treatment sessions, and may be even ineffective in some cases [28].

Surgical resection constitutes another treatment modality for mLM. However, the uncertain border under gross visualization or preoperative imaging often results in inadequate resection, along with potential damage to nerves or vessels [29]. Therefore, the purpose of our study is to identify the margins of mLM lesions intraoperatively based on ICGL, thereby guiding surgical resection combined with sclerotherapy to improve outcomes. Compared with a conventional treatment approach, the experimental group (ICGL-guided partial resection of mLM + penetration sclerotherapy) demonstrated a significantly improved cure rate for mLM, without increase in postoperative complications.

Lymphatic drainage patterns observed in mLM cases in this cohort were characterized by the presence of multiple slender, tortuous, and interwoven lymphatic vessels forming a network within the lesion. This imaging feature highly corresponded with the histological characteristics of microcystic lesions, which consist of numerous tiny lymphatic lumens. The scattered small lymphatic vessels indicated the presence of extensive structural abnormalities of the lymphatic network and impaired lymphatic return dynamics in this type of lesion. This may partially explain the relatively poor response of microcystic lesions to sclerotherapy and their higher propensity for recurrence, and also provided theoretical support for considering the use of targeted anti-lymphangiogenic agents or a more precise surgical resection combined with sclerotherapy. Using ICGL technology, we may distinguish the main body of microcystic lesions with intense fluorescence signals and the surrounding scattered microcysts with sparse fluorescence signals. During the operation, we resected the majority of mLM tissue to achieve maximum “debulking”, while intentionally preserving the peripheral scattered lesions to protect normal tissues. This approach allowed for maximizing curative effect while minimizing iatrogenic injury. Moreover, ICGL-guided partial resection of mLM not only directly removed a substantial portion of the lesion but also precisely delineated the surrounding microcysts, providing positioning information for penetration sclerotherapy.

In our study, a significant difference in cure rate was observed (65.0% vs. 26.3%, *p* < 0.05). Several reasons may explain this finding. First, ICGL navigation delineates the main lesion margin in real time, allowing more complete resection of main lesion compared to experience-dependent surgery. Second, after resection, ICG fluorescence helps identify residual microcysts that are invisible under white light, enabling targeted sclerotherapy. Third, although a learning curve exists, alternating ICGL and control cases minimized its impact. Thus, this synergistic approach potentially enhanced the likelihood of achieving postoperative complete resolution.

Notably, we observed no statistically significant differences between the two groups in subsequent treatment (2 vs. 3, *p* > 0.05). This may be attributed to the limited sample size, or the advantage brought by ICGL had not yet significantly reduced treatment frequency, which requires longer follow-up for verification. In addition, the operative time was relatively prolonged in the experimental group, owing to the intraoperative ICGL procedure. However, with real-time guidance from ICGL, the duration of mLM lesion resection was relatively shortened. The difference in operative time between the two groups was approximately 9 min, and statistical analysis revealed no significant difference (*p* = 0.091). Furthermore, no statistical differences were observed regarding the overall incidence of complications (fever, pigmentation, infection, abnormal hair growth, subcutaneous induration, and scar hyperplasia) (*p* > 0.05). These findings indicated that the real-time, clear visualization of the margin provided by ICGL facilitated more precise intervention by the surgeon without increasing additional surgical risks, thereby reflecting the safety and feasibility of this technique. In addition, after applying the inclusion and exclusion criteria, three cases in each group with coexistence of other vascular malformations were still excluded based on postoperative pathology, indicating that mLM cases cannot be accurately diagnosed solely through physical examination and preoperative imaging during the screening process, which also limited the sample size.

In this research, the actual enrolled sample size in this study was 20 cases in the experimental group and 19 cases in the control group, which was slightly lower than the initially estimated minimum requirement of 20 cases per group. This was mainly due to loss to follow-up and coexistence of other vascular malformations. A post hoc power analysis, based on the actual sample sizes (20 vs. 19) and the observed cure rates (65.0% in the experimental group vs. 26.3% in the control group), revealed that the achieved statistical power for the primary outcome was approximately 75%, which is slightly below the conventional recommended level of 80%. Nevertheless, this value remains close to the acceptable threshold, and a statistical difference between the two groups was observed (*p* < 0.05). Therefore, the reduction of one case in sample size had a limited impact on the study power, and the main conclusion (superiority of the experiment over the control) is robust. However, given that the power did not reach 80%, future studies with larger sample sizes are warranted to validate these findings.

Several limitations of our study deserve comment. First, this was a single-center study with a limited sample size and a potential learning curve for ICG navigation. This limits generalizability to centers with different surgical volumes or less experience, and the findings require multicenter validation. Second, the control group included only 19 cases (vs. the planned 20). Given the significant between-group difference (65.0% vs. 26.3%, *p* < 0.05), this slight shortfall is unlikely to alter the conclusion, but it may modestly overestimate the effect size, so extrapolation to routine practice should be cautious. Third, blinding of surgeons and patients was not possible due to the visible nature of ICG, although outcome assessors were blinded. This limits generalizability to non-ICG-based procedures where double-blinding is feasible; however, the blinded outcome assessment mitigates detection bias. Fourth, both groups received the same sclerotherapy protocol, so the between-group difference can be attributed to the addition of ICGL guidance. However, we cannot determine whether ICGL guidance would be beneficial without concomitant sclerotherapy, nor can we isolate the independent contribution of ICGL-guided sclerotherapy versus ICGL-guided resection. Fifth, the follow-up period is relatively short (median 10–11 months) and varies among patients, and extended follow-up periods were necessary to confirm long-term recurrence rates. Finally, this technique was only applicable to localized lesions, which limited its application in widespread and refractory mLM cases.

## 5. Conclusions

The results from this prospective, randomized clinical trial suggest that ICGL-guided partial resection combined with penetration sclerotherapy may be associated with a higher favorable cure rate in the management of mLM. This strategy delineates the lesional extent by detecting ICG fluorescence signal, thereby enabling both the resection of the main lesion and the precise penetration sclerotherapy of residual microcysts. Our findings further validate the clinical value of the ICGL in guiding targeted therapy for mLM, and its broader clinical application could be confirmed by further research.

## Figures and Tables

**Table 1:** Baseline clinical characteristics of the research subject.

Characteristics	Experimental Group (N = 20)	Control Group (N = 19)	*p* Value
Gender			0.752
Male	11	9	
Female	9	10	
Age (years)			0.748
0~3	9	7	
>3	11	12	
Lesion location			0.667
Cervicofacial region	3	4	
Extremities	11	12	
Trunk	6	3	
Maximum diameter (cm)			0.320
≤5	15	11	
>5	5	8	

**Table 2:** Curative effects comparison between the two groups.

Group	N	Excellent	Good	Fair	Poor	Effective Rate (%)	Cure Rate (%)	Subsequent Treatment
Experimental group	20	13	4	2	1	95.0	65.0 *	2
Control group	19	5	9	4	2	89.5	26.3	3

* *Indicates p < 0.05*.

**Table 3:** Comparison of peri- and postoperative variables between the two groups.

Characteristics	Experimental Group	Control Group	*t*/*Z*	*p* Value
Operative time (minutes)	50.00 ± 15.05	41.53 ± 14.60	1.738	0.091
Postoperative drainage volume (mL)	9.00 (4.00, 18.00)	10.50 (6.00, 24.00)	−0.872	0.383
Follow-up period (months)	11.00 (6.25, 15.00)	10.00 (7.00, 12.00)	−0.411	0.681

**Table 4:** Comparison of postoperative complications between the two groups.

Characteristics	Experimental Group (N = 20)	Control Group (N = 19)	*p* Value
Fever	3	1	0.605
Pigment	1	3	0.342
Abnormal hair growth	3	2	1.000
Local infection	1	1	1.000
Subcutaneous induration	6	7	0.741
Scar hyperplasia	4	2	0.661

## Data Availability

All authors gave their consent for publication of the paper.

## Abbreviations

cLM	Cystic lymphatic malformation
mLM	Microcystic lymphatic malformation
US	Ultrasonography
CT	Computed tomography
MRI	Magnetic resonance imaging
ICGL	Indocyanine green lymphography

## References

[1] Nzelu D., Panayotidis I., Smith G.D., Pandya P. (2024). Fetal Cystic Lymphatic Malformations: Systematic Review on Pregnancy and Neonatal Outcomes. J. Ultrasound Med..

[2] Wiegand S., Wichmann G., Dietz A., Werner J.A. (2023). Association between malformation type, location and functional deficits in lymphatic malformations of the head and neck in children. Eur. Arch. Otorhinolaryngol..

[3] Zhang C., Liu J., Wang Y., Zheng Y., Malashicheva A., Qi L., Shi X., Liu J. (2025). Lymphatic Malformation: Classification, Pathogenesis, and Therapeutic Strategies. Ann. Vasc. Surg..

[4] Marchand A., Caille A., Gissot V., Giraudeau B., Lengelle C., Bourgoin H., Largeau B., Leducq S., Maruani A. (2022). Topical sirolimus solution for lingual microcystic lymphatic malformations in children and adults (TOPGUN): Study protocol for a multicenter, randomized, assessor-blinded, controlled, stepped-wedge clinical trial. Trials.

[5] Usui H., Shinkai M., Tanaka S., Morishima R., Shirane K., Kondo T., Mochizuki K., Kitagawa N. (2025). Novel technique of sclerotherapy for superficial lymphatic malformation. Front. Pediatr..

[6] Sarreau M., Pelluard F., Martin Berenguer S., Sauvestre F., Coatleven F., Cardinaud F., Guibaud L., Collardeau Frachon S. (2025). Extensive Cystic lymphatic malformation with PIK3CA-Related Overgrowth Spectrum: Prenatal Diagnosis and Autopsy Findings in 3 Fetal Cases. Fetal Pediatr. Pathol..

[7] Petkova M., Ferby I., Makinen T. (2024). Lymphatic malformations: Mechanistic insights and evolving therapeutic frontiers. J. Clin. Investig..

[8] Leboulanger N., Bisdorff A., Boccara O., Dompmartin A., Guibaud L., Labreze C., Lagier J., Lebrun-Vignes B., Herbreteau D., Joly A. (2023). French national diagnosis and care protocol (PNDS, protocole national de diagnostic et de soins): Cystic lymphatic malformations. Orphanet J. Rare Dis..

[9] Schreiber A., Soupre V., Kadlub N., Galliani E., Picard A., Chretien-Marquet B., Pannier S., Guero S., Khen-Dunlop N., Hadj-Rabia S. (2019). Does surgery of lymphatic malformations lead to an increase in superficial lymphangiectasia? A retrospective study of 43 patients. Br. J. Dermatol..

[10] Parashar G., Shankar G., Sahadev R., Santhanakrishnan R. (2020). Intralesional Sclerotherapy with Bleomycin in Lymphatic Malformation of Tongue an Institutional Experience and Outcomes. J. Indian Assoc. Pediatr. Surg..

[11] Gilat E.K., Cohen I., Brin D., Greenberger S., Raskin D. (2024). A 14-year single-center experience evaluating sclerotherapy efficacy in lymphatic malformations. J. Vasc. Surg. Venous Lymphat. Disord..

[12] Lee E., Biko D.M., Sherk W., Masch W.R., Ladino-Torres M., Agarwal P.P. (2022). Understanding Lymphatic Anatomy and Abnormalities at Imaging. Radiographics.

[13] Da Ros V., Iacobucci M., Puccinelli F., Spelle L., Saliou G. (2018). Lymphographic-Like Technique for the Treatment of Microcystic Lymphatic Malformation Components of <3 mm. AJNR Am. J. Neuroradiol..

[14] Ding Y., Sun W., Wang H., Wang L., Wei H., Hou G., Zhang X., Zhang W. (2021). Successful Treatment of Vaginal and Pelvic Microcystic Lymphatic Malformation with 50% Ethanol Injection via Lymph Node Angiography. J. Pediatr. Adolesc. Gynecol..

[15] Yoshida S., Imai H., Roh S., Mese T., Koshima I. (2023). Cystic Lymphatic Malformation with Lymphedema Treated by Lymphaticovenular Anastomosis Combined with Ethanol Sclerotherapy. Plast. Reconstr. Surg. Glob. Open.

[16] Tripke V., Sommer N. (2023). An update on liver surgery-a new terminology and modern techniques. Innov. Surg. Sci..

[17] Sakai H., Miyazaki T., Tsukuura R., Yamamoto T. (2025). The Upper Arm Lymphosome: Watershed of Upper Arm Lymphatic Pathways Evaluated with Indocyanine Green Lymphography. Lymphology.

[18] Han T., Shi L., Huang S., Shen W. (2025). Application value of indocyanine green lymphography in the management of stage I/IIA microcystic lymphatic malformation of the tongue. Head Face Med..

[19] Han T., Ye D., Cui J., Huang S., Shen W. (2025). Impact of Intracystic Hemorrhage on Therapeutic Outcomes in Macro/Mixed Cystic Lymphatic Malformation: A Retrospective Cohort Study. Children.

[20] Alqutub A., Baamir N.J., Mofti Z., Zawawi F., Al-Khatib T. (2024). Sclerotherapy vs. surgical excision for lymphatic malformations of the head and neck: A systematic review and meta-analysis. Eur. Arch. Otorhinolaryngol..

[21] De Maria L., De Sanctis P., Balakrishnan K., Tollefson M., Brinjikji W. (2020). Sclerotherapy for lymphatic malformations of head and neck: Systematic review and meta-analysis. J. Vasc. Surg. Venous Lymphat. Disord..

[22] Xie C., Guo Z., Lin W., Wang P., Yang W., Wang H. (2024). Single-Site Endoscopic Surgery for Soft Tissue Lesions: An Innovative Technique. Cureus.

[23] Berenstein A., Bazil M.J., Sorscher M., Blei F., De Leacy R., Shigematsu T., Waner M., Fifi J.T. (2023). Percutaneous sclerotherapy of microcystic lymphatic malformations: The use of an innovative gravity-dependent technique. J. Neurointerv. Surg..

[24] Mack J.M., Peterson E.C., Crary S.E., Moran J.H., Neville K., Pierce C.D., Richter G.T. (2022). Pharmacokinetics of bleomycin sclerotherapy in patients with vascular malformations. Pediatr. Blood Cancer.

[25] Markovic J.N., Nag U., Shortell C.K. (2020). Safety and efficacy of foam sclerotherapy for treatment of low-flow vascular malformations in children. J. Vasc. Surg. Venous Lymphat. Disord..

[26] Moreno-Alfonso J.C., Triana P., Miguel Ferrero M., Diaz Gonzalez M., Lopez Gutierrez J.C. (2024). Risk factors for sequelae after surgery for lymphatic malformations in children. J. Vasc. Surg. Venous Lymphat. Disord..

[27] Cha J.G., Lee J., Lee S.Y., Chung H.Y., Lee S.J., Huh S., Kim J.Y., Hong J. (2022). Safety and Efficacy of Bleomycin Slow Infusion Sclerotherapy Using a Syringe Pump for Microcystic and Mixed Lymphatic Malformations. Cardiovasc. Interv. Radiol..

[28] Hyvonen H., Salminen P., Kyrklund K. (2022). Long-term outcomes of lymphatic malformations in children: An 11-year experience from a tertiary referral center. J. Pediatr. Surg..

[29] Poget M., Fresa M., El Ezzi O., Saliou G., Doan M.T., de Buys Roessingh A. (2022). Lymphatic malformations in children: Retrospective review of surgical and interventional management. Pediatr. Surg. Int..

